# How a generally well-accepted measles vaccine mandate may lead to inequities and decreased vaccine uptake: a preregistered survey study in Germany

**DOI:** 10.1186/s12889-022-14075-y

**Published:** 2022-10-03

**Authors:** Julia Neufeind, Nora Schmid-Küpke, Eva Rehfuess, Cornelia Betsch, Ole Wichmann

**Affiliations:** 1 Immunization Unit, Robert Koch-Institute, Berlin, Germany; 2 Institute for Medical Information Processing, Biometry and Epidemiology, Ludwig-Maximilians-Universität, Munich, Germany; 3 Pettenkofer School of Public Health, Munich, Germany; 4 Center for Empirical Research in Economics and Behavioral Sciences (CEREB), University of Erfurt, Erfurt, Germany; 5 Media and Communication Science, University of Erfurt, Erfurt, Germany; 6 Bernhard Nocht Institute for Tropical Medicine, Hamburg, Germany

**Keywords:** Vaccine hesitancy, Mandate, Compulsory, Measles, Reactance, Health policy

## Abstract

**Background:**

In Germany, a measles vaccine mandate came into effect in March 2020, requiring proof of measles immunization for children attending kindergarten or school and for staff in a variety of facilities. Mandates can be successful if implemented with care and in a context-sensitive manner. They may, however, also lead to inequities and decreased uptake of other vaccines. The aim of this study was to investigate the acceptance and potential unintended consequences of the measles vaccine mandate in Germany.

**Methods:**

As part of a larger evaluation project on the new mandate, we conducted an online survey among parents in August/September 2020. We assessed differences in knowledge about the mandate and the measles vaccine by socio-economic status. We used linear and logistic regression to estimate how reactance to the mandate was associated with vaccination status and vaccination intention against other diseases. We used mediation analysis to measure how trust in institutions had an impact on the attitude towards the mandate, mediated by level of reactance.

**Results:**

In total, 4,863 parents participated in the study (64.2% female, mean age = 36.8 years). Of these, 74.1% endorsed a measles vaccine mandate for children. Parents with lower socio-economic status had less knowledge about the mandate and the measles vaccine. The higher parents’ levels of reactance, the lower the vaccination intentions and the likelihood for the child to be vaccinated against other diseases. Furthermore, higher institutional trust decreased the level of reactance and increased positive attitudes towards the mandate (partial mediation).

**Conclusions:**

The new measles vaccine mandate in Germany, though well accepted by many, might have unintended consequences. Parents with lower socio-economic status, who know less about the mandate and vaccine, might be less likely to comply with it. The mandate may also lead to some parents omitting other childhood vaccines, as a way to restore their freedom. This could decrease vaccination coverage of other vaccines. Any potential loss of trust might provoke more reactance and lower acceptance of mandates. Policymakers should now expand communication activities on the mandate, monitor trends in vaccination coverage carefully and take measures to strengthen trust.

**Supplementary Information:**

The online version contains supplementary material available at 10.1186/s12889-022-14075-y.

## Introduction

Germany has struggled for a long time to eliminate measles [1]. Vaccination coverage was insufficient to prevent transmission and outbreaks in several but not all federal states [2, 3]. In 2019, more than 430 autochthonous measles cases were notified in Germany resulting in an incidence of 5.2 per 1 million inhabitants [4]. Educative measures were regarded as insufficient to further increase vaccine coverage [5–7]. In response, in March 2020, in the beginning of the COVID-19 pandemic, a new selective measles vaccine mandate was introduced in Germany – all other immunizations remaining voluntary [8]. The new measles mandate requires all children in preschool childcare and schools as well as staff in a variety of facilities to provide proof of their immunization against measles. Proof has to be provided immediately by those who enter childcare or school after March 2020, for children already in care a transition period until July 2021 was determined, which was later extended to July 2022 due to the COVID-19 pandemic. Potential sanctions include the rejection of access to preschool childcare, penalties up to EUR 2,500, or inability to sign a new job contract in relevant facilities. Exemptions exist for individuals with medical contraindications or naturally acquired immunity.

There is evidence that vaccine mandates can increase vaccination coverage [9]. For example, France, Australia and Italy saw an increase in vaccine uptake after recent extensions of different vaccine mandates [10–13], in the United States mandates considerably increased influenza vaccination coverage among healthcare workers [14]. Omer et al. emphasize that vaccine mandates can be successful when implemented with care and in a context-sensitive manner [15]. Mandates may, however, also have unintended consequences, both ethical and practical [16, 17], which one needs to examine carefully and identify appropriate countermeasures.

First, a mandate might not affect all population groups equally. Studies show that a mandate may lead to or increase inequities [18]. Ethics councils in Germany and Great Britain as well as the World Health Organization (WHO) have expressed their worry that a vaccine mandate may compound structural disadvantages, especially among marginalized groups [17, 19, 20], who might also be more prone to exhibit institutional mistrust and hence more apprehension towards a mandate. As a prerequisite for getting vaccinated, those affected by the mandate should know about the regulations in place, about the vaccine and its effectiveness as well as potential side effects. It would thus be an inequity if a lack of knowledge disproportionately affected those with lower socio-economic status (education, income).

Experimental evidence also suggests that mandates may evoke reactance [21] and lead to counter-action, such as omitting other voluntary vaccines or protesting against the policy [22]. Individuals might seek to restore their restricted freedom by performing a behavior similar to the threatened behavior, i.e., non-vaccination, (“related boomerang effects”) [23]. A behavioral experiment in Germany found that a selective mandate (requiring only one specific vaccine while the rest remain voluntary) increased the level of reactance for individuals with a rather negative vaccination attitude [24]. This led to a decrease in vaccination uptake when participants had to decide about a second voluntary vaccination. The authors replicated these results in a similar experimental study [25] and a survey study [26], both in German and US samples.

Mandates are introduced into a pre-existing social and political context. Whether or not mandates are favored and whether they spark reactance may also depend on institutional trust. In Germany, 60% of the population indicated to trust in the national government when the mandate was introduced in 2020, at the beginning of the pandemic; trust declined over the past two years to 48% in 2022 [27–30]. In the last twenty years institutional trust in Germany fluctuated, but levels were similar at the beginning and end of the period [31]. Coercive measures, as they affect our freedom of choice, demand that people agree that they are necessary or that they trust in those who believe they are necessary [32]. This is even more true when dealing with sensitive aspects such as the basic right of the child to life and physical integrity, and the rights of parents [33, 34]. When individuals trust in societal and political institutions, they tend to believe that politics treats them justly and represents them well [35]. In a climate of high institutional trust, reactance due to a new selective mandate might be less prominent and the attitudes towards a mandate more positive.

The mixed evidence on the effectiveness and potential unintended consequences of mandates lay bare the need for a good evaluation of any newly introduced vaccine mandate. To evaluate the acceptance and potential unintended consequences of the measles vaccine mandate in Germany, we conducted a set of studies and data analyses to cover a broad range of research questions. The present paper draws upon cross-sectional, observational data and looked at parents of children 0–18 years. More specifically, in this study we aimed to evaluate the reaction of parents to the new measles vaccine mandate and to understand whether.

Hypothesis 1: Lower socio-economic status (education and income) is a risk factor for less knowledge about the measles vaccine mandate and less knowledge about the measles vaccine among parents;

Hypothesis 2: Higher reactance to the measles mandate among parents decreases uptake of other vaccines and the intention to vaccinate children against other diseases; and

Hypothesis 3: Parents with lower institutional trust feel more reactance and consequently have a more negative attitude towards the measles mandate.

## Methods

### Study population and recruitment

Data were collected as part of a broader prospective longitudinal study evaluating the introduction of the measles vaccine mandate in Germany, with six data collections over a period of 20 months. The data included in this study are from the first data collection point in August/September 2020, 6 months after the introduction of the measles mandate. We recruited parents with at least one child aged 0–18 years. We oversampled parents of children aged 0–2 years, as the measles vaccine mandate demands one dose of measles vaccine for children at the age of 1 year and two doses at the age of two years [36]. Hence, we wanted to include enough participants for whom the decision about whether or not to vaccinate their child against measles was due or had just been taken. We initially aimed to recruit at least 2,200 parents of children aged 0–2 years and 2,200 parents of children aged 3–18 years. Participants were recruited from a non-probabilistic German sample (online access panel). We used pre-existing information from the panel provider on whether the individual had children and their respective age and invited parents of children aged 0–18 via e-mail to participate in the survey. For parents of children aged 3–18 years the sample was quota-representative for gender, region, education and age [37]. The panel provider could not offer quota control for parents of children aged less than 3 years. The survey was conducted as an online survey via VOXCO edition 6.0.0.51. The ethics commission of the Berlin Medical Chamber (#Eth-17/22) and the data protection officer at the Robert Koch Institute approved the survey. Participants were informed about the aims of the study and provided their informed consent online before starting the survey. There was no study intervention and the anonymity of the participants was guaranteed at all times.

### Survey instrument

All items used (translated into English) and the R code can be found at https://osf.io/yfect/?view_only=04b0f6538b3e4d8484541534800e7ef5. Hypotheses were preregistered (AsPredicted https://aspredicted.org/nq45k.pdf). We asked parents to answer all questions with regards to their youngest child only, in order to be unambiguous in case participants had several children and to increase the chance that the child meets the inclusion criteria. The constructs were assessed in the following reported order.

#### Sociodemographics

For socio-economic status we assessed level of education and level of income. For education we applied the International Standard Classification of Education (ISCED) [38]. ISCED considers both the highest school level completed and the highest professional qualification attained. We subsumed ISCED 1 and 2 to “low education,” ISCED 3 and 4 to “medium education” and ISCED 5 and 6 to “high education.” We assessed income using the “OECD-modified scale” [39]. The equivalence scale considers household size and weighs the age of the respective household members. We calculated terciles where the lower tercile is “low income,” the middle tercile is “medium income” and the highest tercile is “high income.” Further sociodemographic variables collected included age, gender, region (i.e., eastern or western Germany) and the age of the respective child.

#### Measles immune status

We assessed measles immune status of the respective child (i.e., vaccination status, number of vaccine doses given, natural immunity).

#### Attitude towards the mandate

We measured the attitude towards the mandate using 5 self-developed items varying according to the groups affected by the mandate, e.g., “The measles vaccine should be mandatory for children going to school and kindergarten.” Items were developed by experts in immunization and health psychology and adapted from a former study [40]. For each of these items, respondents stated their level of agreement on a 5-point Likert scale ranging from “strongly disagree” (score = 1) to “strongly agree” (score = 5). We calculated a mean score, ranging from negative (score = 1) to positive attitude (score = 5) towards the mandate, Cronbach’s alpha = 0.91.

#### Reactance

We assessed reactance triggered by the mandate. Reactance is defined as a motivation to restore freedom of choice after restrictions [41]; it is prompted when an individual perceives any of his or her free choices to be threatened [21]. It can be defined as a trait but also as a state, e.g., reactance can be triggered by a coercive measure [42]. We drew upon a validated scale for state reactance consisting of 4 items, (e.g., “Are you frustrated about the measles vaccine mandate?”) [43]. For each of these items, respondents stated their level of agreement on a 5-point Likert scale ranging from “not at all” (score = 1) to “very much” (score = 5). We calculated a mean reactance score ranging from low reactance (score = 1) to high reactance (score = 5), Cronbach’s alpha = 0.95. For descriptive analysis we interpreted mean reactance score values 1—< 2.5 as (rather) not reactant, 2.5 – 3.5 as undecided and > 3.5 – 5 as (rather) reactant; these cut-offs respect the intervals of the 5-point Likert scale, with score = 3 ± 0.5 as the middle category “undecided” with equal distance to the upper and lower category.

#### Knowledge about the measles vaccine mandate

We assessed knowledge about the measles vaccine mandate with 5 questions, e.g., “To prove immunity against measles a positive blood test for antibodies is sufficient” and calculated a sum score ranging from no correct answer (score = 0) to all answers correct (score = 5). Items were self-developed by experts in immunization and health psychology. We counted “don’t know” answers as not correct.

#### 5C psychological determinants

We further included the 5C psychological antecedents of vaccination as covariates to control for other factors that would affect vaccination behavior – the 5C is a widely used scale to explain vaccination behavior [44]. The 5C measure not only confidence in vaccines and the system that delivers them, constraints (structural and psychological barriers), and complacency (not perceiving disease as high risk) but also collective responsibility (willingness to protect others) and calculation (engagement in extensive information searching) and can be regarded as more comprehensive than the 3C initially developed by WHO [45]. We applied the 5C short scale that comprises five items, one for each construct (confidence, collective responsibility, constraints, complacency and calculation), e.g., “I am completely confident that vaccines are safe.” For each of these items the respondents stated their level of agreement on a 5-point Likert scale ranging from “strongly disagree” (score = 1) to “strongly agree” (score = 5).

#### Knowledge about the measles vaccine

We measured knowledge about the measles vaccine with 7 questions (3 items adapted specifically to measles from Zingg & Siegrist [46], 4 items self-developed by experts in immunization and health psychology), e.g., “The measles vaccine increases the occurrence of allergies.” We built a sum score ranging from no correct answer (score = 0) to all answers correct (score = 7). “Don’t know” answers were handled as not correct.

#### Other vaccination behaviors

We assessed whether parents intended to vaccinate or had vaccinated their youngest child according to the recommendation of the National Immunization Technical Advisory Group (NITAG) in Germany [47]. Parents of a child from the age of 2 months to under 1 year were asked about their child’s vaccination status for pneumococcal and hexavalent diphtheria, tetanus, pertussis, *Hemophilus influenzae* type b, poliovirus, hepatitis B combination vaccine (dichotomous variable, “not vaccinated” = 0, “vaccinated” = 1; the NITAG recommends routine immunization against these diseases under the age of 1). Parents were also asked about their vaccination intention for meningococcal C vaccine (the NITAG recommends meningococcal C vaccination at 12 months of age). Parents of children aged 1–9 years were asked whether they intended their child to have the human papillomavirus (HPV) and tetanus, diphtheria, pertussis (Tdap) booster vaccine (the NITAG recommends the HPV vaccine for adolescents 9–14 years and Tdap booster vaccine for adolescents 9–16 years). Vaccination intention was assessed on a 5-point Likert scale ranging from “definitely not vaccinate” (score = 1) to “definitely vaccinate” (score = 5).

#### Institutional trust

We assessed generalized institutional trust, e.g., how much trust people had in institutions such as the police or the national government [35]. Respondents stated their level of trust on a 5-point Likert scale ranging from “no trust” (score = 1) to “very much trust” (score = 5). We calculated a mean score “institutional trust” consisting of 5 items, Cronbach’s alpha = 0.88.

### Statistical analyses

Analyses were conducted in R [48, 49]. Complete cases analysis was pursued for all items, as our sample size was large enough for our analysis and little data was missing (7.0% of data was missing in the income equivalence scale, 0.8% in the reactance score, 2.7% in the institutional trust score, 12.8% in the pneumococcal vaccination status, 3.5% in the hexavalent vaccination status, data in all remaining variables was complete). For Hypothesis 1 we conducted separate one-way ANOVAs to compare the effect of level of education (low, medium and high education) and level of income (low, medium and high income) on knowledge about the measles vaccine mandate and on knowledge about measles vaccine, respectively. We conducted post hoc comparisons using the Tukey HSD test.

For Hypothesis 2 we conducted the analyses on two target populations, defining a subset of parents with a child aged >= 2 months to < 1 year and a subset of parents with a child aged 1–9 years. We performed groupwise multiple logistic and linear regressions to assess the association between level of reactance and vaccination status and vaccination intention, respectively. As an additional analysis (not preregistered), guided by a directed acyclic graph (DAG) (see Supplementary Fig. 1), we included the 5C psychological determinants of vaccination as covariates to control for other major psychological factors that influence vaccine decision-making [25, 44]. We then included institutional trust, which we hypothesized would have an influence both on reactance and vaccination behavior [50]. We report odds ratios (OR) and β estimates, 95% confidence intervals (CI), and R2 or Nagelkerkes R2, area under the curve (AUC) and Akaike information criterion (AIC) to assess model fit. We computed variance inflation factors (VIFs) to test for multicollinearity and interpreted values < 5 as presenting no multicollinearity issues.

For Hypothesis 3 we performed a mediation analysis to test whether reactance mediated the effect of institutional trust on the attitude towards the mandate.

As preregistered, we repeated all analyses including age, region and gender as potential confounders [3, 51–53].

## Results

In total, 4,863 parents participated in our study (i.e., at a first point of data collection 6 months after introduction of the measles mandate). Of these 3,126 were female, the mean age was 36.8 (SD = 8.0) years; 3,459 parents had a youngest child affected by the measles vaccine mandate (i.e., a child in preschool childcare or school where the mandate is enforced). Among 2,500 parents with their youngest child aged 0–2 years, 1,149 (46.0%) reported that their child went to kindergarten; among 678 parents with their youngest child aged 3–5 years, 625 reported that their child went to kindergarten (92.2%). We assumed that all children aged 6 years and older in our sample (*n* = 1,685) went to school, as schooling is compulsory in Germany. For further details see Table 1.

**Table 1:** Characteristics of study population

		**Study population 2020**		**Population in Germany 2019 (in million)**
		**Parents of children aged 0–35 months**	**Parents of children aged 36 months – 18 years**	**Parents of children aged 2–17 years **[37]
n		2,500	2,363	16.0
Age (years): n (%)	18–29 30–39 40–49 50–59 > = 60	675 (27.0) 1,518 (60.7) 288 (11.5) 16 (0.0) 3 (0.0)	178 (7.5) 923 (39.1) 915 (38.7) 318 (13.5) 29 (1.2)	2.4 (15.2) 5.7 (35.5) 5.9 (37.1) 1.9 (11.8) 0.6 (< 1)
Gender: n (%)	Male Female	705 (28.2) 1,795 (71.8)	1,032 (43.7) 1,331 (56.3)	7.5 (46.7) 8.5 (53.2)
Education (highest school degree): n (%)	High Medium Low	1,481 (59.2) 840 (33.6) 179 (7.2)	915 (38.7) 913 (38.6) 535 (22.7)	6.1 (38.1) 6.3 (39.3) 3.6 (22.6)
Region: n (%)	East West	545 (21.8) 1,955 (78.2)	469 (19.8) 1,894 (80.2)	2.8 (17.7) 13.2 (83.3)
Age of child in years: mean (SD)		1.0 (0.8)	9.3 (4.7)	-
Number of children: mean (SD)		1.7 (0.9)	1.8 (0.9)	-

**Fig. 1:**
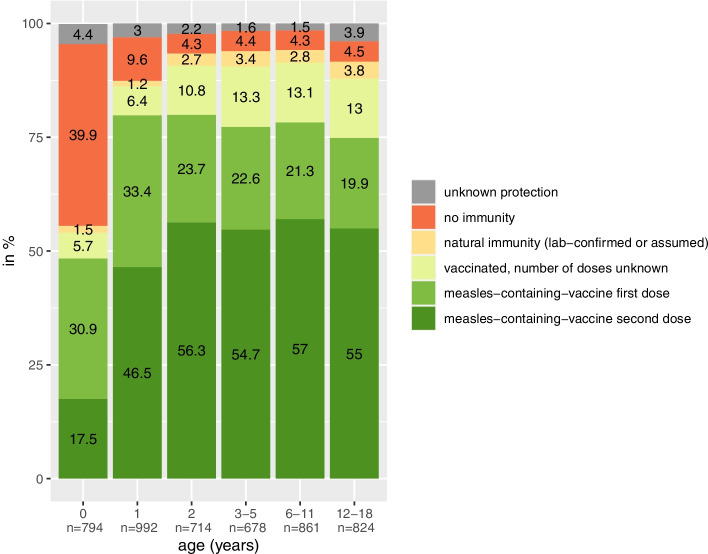
Measles protection for different age groups according to parents reporting on their youngest child

Figure 1 displays the measles immune status of children in different age groups in our sample (self-reporting of parents). By definition of the mandate, children are regarded to be fully vaccinated if they have received two doses of measles vaccine by the age of two or they are exempted from vaccination if they have acquired natural immunity. In all age groups a considerable proportion of parents reported incomplete vaccination, non-vaccination or unclear vaccination status of their youngest child. This implies that the new vaccine mandate will affect them directly.

Parents had a predominantly positive reaction to the measles vaccine mandate (i.e., a rather positive attitude towards the mandate, low level of reactance): 74.1% of parents rather agreed that the measles vaccine should be mandatory for children going to school and kindergarten, 13.8% were undecided and 12.2% rather disagreed; 74.3% of parents were rather not reactant (reactance score 1 – < 2.5), i.e., they were rather not frustrated, annoyed or disturbed about the mandate, 11.8% were undecided (reactance score 2.5 – 3.5), and 14.0% were rather reactant (reactance score > 3.5 – 5).

**Fig. 2:**
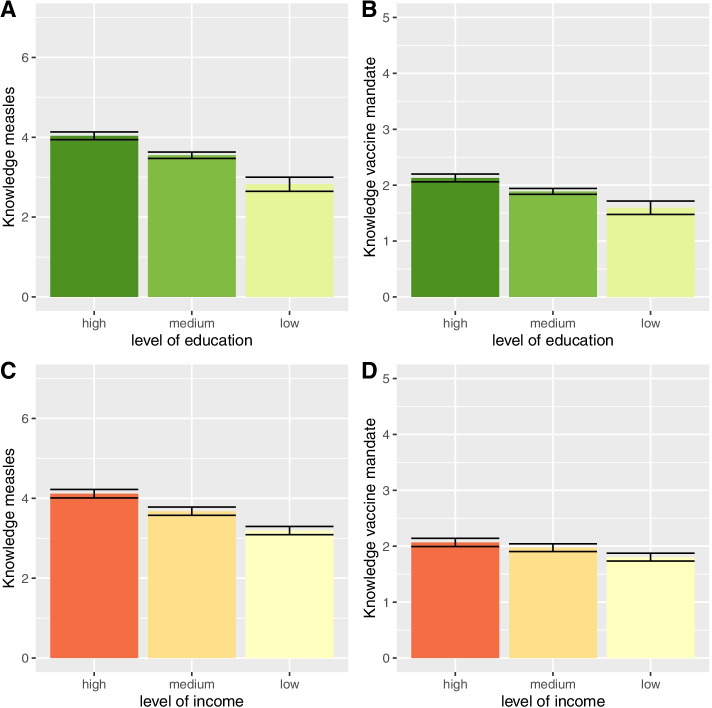
Knowledge about the measles vaccine and the mandate in parents by socio-economic status. Notes: Knowledge about the measles vaccine = Score, Range 0–7. Knowledge about the measles vaccine mandate = Score, Range 0–5. Error bars = 95% CI. Education = ISCED classification [38], Income = OECD equivalence scale [39]

For Hypothesis 1 we aimed to analyze differences in knowledge about the measles vaccine mandate and the measles vaccine by socio-economic status (level of education and level of income). As can be seen in Fig. 2A and B, there was a significant positive effect of level of education on knowledge about the measles vaccine [F(2, 4860) = 73.93, *p* =  < 0.001] and on knowledge about the measles vaccine mandate [F(2, 4860) = 32.05, *p* =  < 0.001]. Post hoc comparisons using the Tukey HSD test indicated significant differences for all groups. The same pattern was observed for level of income: there was a significant effect of level of income on knowledge about measles [F(2, 4520) = 77.45, *p* =  < 0.001] (Fig. 2C) and on knowledge about the measles vaccine mandate [F(2, 4520) = 27.33, *p* =  < 0.001] (Fig. 2D). Post hoc comparisons using the Tukey HSD test indicated that individuals with low income on average knew less about the measles vaccine mandate (*p* < 0.001) and about measles (*p* < 0.001) than parents with high income. Individuals with low income also knew less about measles (*p* = 0.003) and the mandate (*p* < 0.001) than parents with medium income. When further adding region, gender and age to the model, the effects remained stable (see Supplementary Table 1 and Table 2).

**Table 2:** Relationship between reactance and vaccination behavior. Results from multiple logistic regression model

*Explanatory variables*	**Hexavalent vaccine**		**Pneumococcal vaccine**	
	*Odds Ratios*	*Odds Ratios*	*Odds Ratios*	*Odds Ratios*
(Intercept)	25.61 (16.42;41.15)	2.00 (0.27;15.03)	16.67 (11.07;25.71)	1.91 (0.27;13.48)
Reactance	**0.50 (0.42;0.59)**	**0.73 (0.58;0.91)**	**0.50 (0.42–0.59)**	**0.69 (0.55;0.86)**
Confidence		**1.52 (1.18;1.96)**		**1.41 (1.11;1.79)**
Collective Responsibility		1.16 (0.88;1.54)		1.20 (0.91;1.58)
Constraints		1.02 (0.80;1.32)		1.09 (0.86;1.39)
Complacency		**0.71 (0.54;0.94)**		0.83 (0.64;1.09)
Calculation		1.12 (0.90;1.40)		0.93 (0.76;1.14)
Observations	671	671	607	607
R^2^ Tjur	0.123	0.166	0.145	0.177
AUC	0.834	0.861	0.733	0.760
AIC	404.7	396.7	437.9	433.4

Blockwise inclusion of covariates

Bold denotes significance at *p* < 0.05

Next, we looked at parental vaccination decisions for childhood vaccines that had just become due or were soon to come according to NITAG recommendations (see Supplementary Table 3). A relevant proportion of children aged >= 2 months up to < 1 year had – according to parental reporting – not yet received the hexavalent and pneumococcal vaccine. The intention to vaccinate against different diseases in the future varied: Tdap booster was much more accepted (83.7%) than the meningococcal C vaccine (69.5%), which in turn was more accepted than the HPV vaccine (48.0%).

**Fig. 3:**
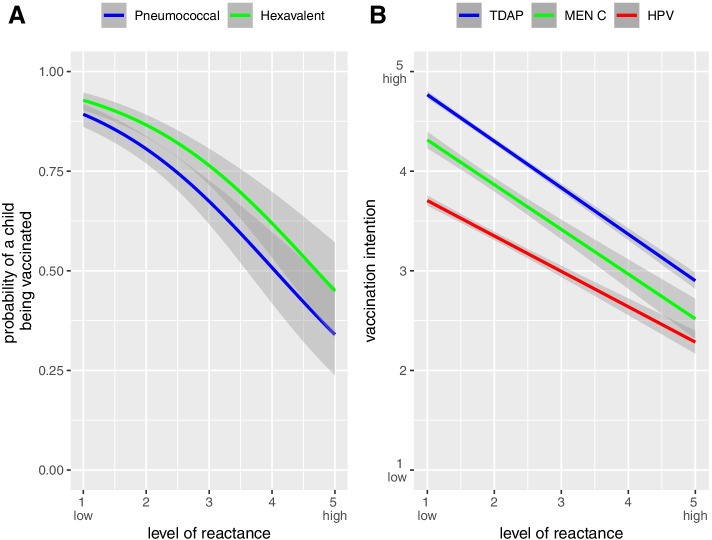
Relationship between level of reactance and likelihood to be vaccinated (**A**) and vaccination intention (**B**). Notes: Estimates are not adjusted; gray bands indicate the 95% confidence intervals. Figure **A** = Results from two logistic regressions, i.e., probability of a child having been vaccinated with pneumococcal (blue curve) or hexavalent vaccine (green curve) by level or reactance. Figure **B** = Results from three linear regressions, i.e., HPV (red curve), meningococcal C (green curve) and TDAP vaccination intention (blue curve) by level of reactance

To test Hypothesis 2, we analyzed whether a higher level of reactance was associated with a lower vaccine uptake and a lower vaccination intention. We conducted a series of stepwise regressions predicting the child’s vaccination status for the hexavalent and pneumococcal vaccines (Fig. 3A, Table 2). Parents’ higher reactance towards the measles vaccine mandate decreased the likelihood for the child to be vaccinated. This means, the greater the parental reactance about the mandate, the less likely parents were to decide to vaccinate their child against vaccines other than measles. The odds ratios decreased but remained significant when the 5C psychological antecedents were added to the model. This implies that even when controlling for the main psychological factors that affect vaccination behavior (the 5C), reactance to the measles mandate still had a relevant effect on parental vaccination behavior for other vaccines. When adding region, gender and age as well as institutional trust to the model, the effects remained stable as well (see Supplementary Table 4). We did not identify relevant multicollinearity in our models.

In addition, we conducted another series of stepwise regressions predicting the parental intention to vaccinate their child with the HPV, Tdap or Men C vaccine (Fig. 3B, Table 3). For all vaccines we found the same pattern: when reactance was higher, the vaccination intention was lower. For example, for the Tdap vaccine, for every two units of greater reactance (for example from “1 = not at all angry” to “3 = undecided”), the vaccination intention was reduced by one unit (for example from “5 = definitely vaccinate” to “4 = rather vaccinate”). The coefficients for reactance decreased, when the 5C psychological antecedents were added to the model, but they remained significant. In short, even when controlling for the main psychological factors that determine vaccination behavior (the 5C), reactance due to the measles mandate still lowered parental vaccination intention for other vaccines. When further adding gender, age and region as well as institutional trust to the model, the effects remained stable (see Supplementary Table 5). There was no multicollinearity in our models.

**Table 3:** Relationship between reactance and vaccination intention. Results from multiple linear regression model

*Explanatory variables*	**HPV vaccine**		**Tdap vaccine**		**Men C vaccine**	
	*Estimates*	*Estimates*	*Estimates*	*Estimates*	*Estimates*	*Estimates*
(Intercept)	4.06 (3.98;4.14)	2.55 (2.15;2.95)	5.23 (5.18;5.29)	3.46 (3.18;3.74)	4.76 (4.63;4.89)	3.44 (2.76;4.12)
Reactance	**-0.36 (-0.39;-0.32)**	**-0.16 0.20;-0.11)**	**-0.47 (-0.49;-0.44)**	**-0.25 (-0.28;-0.22)**	**-0.45 (-0.51;-0.39)**	**-0.29 (-0.37;-0.20)**
Confidence		**0.35 (0.31;0.39)**		**0.24 (0.21;0.27)**		**0.27 (0.19;0.35)**
Collective Responsibility		0.00 (-0.05;0.06)		**0.11 (0.07;0.15)**		0.01 (-0.09;0.11)
Constraints		**0.05 (0.01;0.09)**		0.02 (-0.01;0.05)		0.06 (-0.02;0.13)
Complacency		**-0.08 (-0.13;-0.02)**		-**0.14 (-0.18;-0.11)**		-0.09 (-0.18;0.01)
Calculation		-0.02 (-0.06;0.01)		**0.08 (0.06;0.11)**		0.02 (-0.04;0.08)
Observations	2872	2872	2872	2872	781	781
R^2^ / R^2^ adjusted	0.123 / 0.123	0.200 / 0.198	0.324 / 0.324	0.417 / 0.416	0.219 / 0.218	0.273 / 0.267
AIC	8930.7	8677.5	6964.1	6550.4	2180.8	2134.9

Blockwise inclusion of covariates

Bold denotes significance at *p* < 0.05

**Fig. 4:**
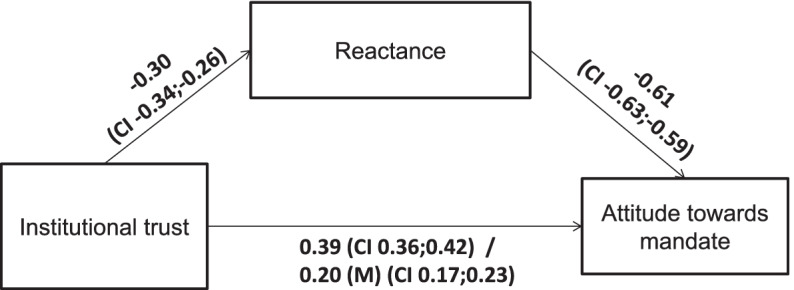
Mediation analysis. Note: All coefficients are β coefficients. Bold denotes significant at *p* < 0.05. The path coefficients after the slash indicate the relation between institutional trust and attitude towards mandate controlled for reactance. (M) indicates a significant mediation effect

For Hypothesis 3 we tested whether reactance mediates the effect of institutional trust on attitude towards mandates (Fig. 4). Our mediation model included institutional trust as the predictor variable, reactance as the mediator variable, and attitude towards the mandate as the outcome variable. Higher institutional trust was significantly and positively related to a more positive attitude towards the mandate, which was partially mediated by the level of reactance (average causal mediation effect [ACME]: β = 0.19, 95% CI = 0.17;0.21, *p* < 0.05). Higher institutional trust was related to less reactance and less reactance was related to a more positive attitude towards the mandate. Thus, a partial mediation effect occurred. When further adding gender, age and region to the model, the effects remained stable (see Supplementary Table 6).

## Discussion

With this study we contribute to the evaluation of the new measles vaccine mandate in Germany and thus the discussion on the effects of mandatory vaccination policies more broadly. The direct ratings of the policy show that the mandate was well accepted by many at the time of its introduction and rejected by some, which suggests a relatively smooth introduction. However, going beyond these direct ratings, the data also suggest that the policy might have unintended consequences. The mandate might disproportionately affect those with lower socio-economic status – who know less about the mandate and the vaccine – and it might have a negative impact on the uptake of other vaccines. Our findings further support the assumption that while a mandate is a relatively straightforward policy, this “simple intervention” is introduced into the “complex system” of German society and interacts with this: we found institutional trust as a prerequisite for lower reactance and a more positive attitude towards the mandate.

Our data confirm that there is a significant proportion of parents whose children are directly affected by the measles vaccine mandate, as they do not have sufficient measles protection yet and will need to get vaccinated under the new mandate. This is in line with official German data on measles vaccination gaps among children [51]. Further evaluation has to show whether the mandate will succeed in closing these vaccination gaps. The majority of participating parents were in favor of the measles vaccine mandate. Still, a relevant proportion had a negative attitude towards it. Also, while reactance to the mandate was generally low, a relevant proportion felt angry about the new mandate. Longitudinal analysis on the basis of the current data will show whether, and if so, how the attitude towards the mandate and the level of reactance will change over time; it could, for example, be that parents get used to the mandate and reactance weakens over time [54].

Our findings on socio-economic differences in knowledge about both the mandate and the measles vaccine (Hypothesis 1) revealed potential unintended consequences: half a year after the introduction of the mandate, parents with low socio-economic status knew less about the mandate and the measles vaccine itself compared to parents with medium and high socio-economic status. This is true for both education and income as domains of socio-economic status and points to a potential inequity. Citizens should be equally well informed about the measles vaccine mandate and the measles vaccine, because those who do not follow the law will experience sanctions. Part of this shortfall might be explained by the pandemic: in Germany, communication activities around the new measles vaccine mandate were initiated shortly before the mandate came into effect in March 2020 – consisting mainly of the establishment of a central website www.masernschutz.de. However, activities were displaced by the COVID-19 pandemic. Differences might also be explained by the fact that generally in Europe, individuals with lower socio-economic status have lower health literacy, that is they find it more difficult to assess, understand, appraise and apply health-related information, which is partly due to skills of the individual and partly due to demands of the system [55, 56]. In Germany, individuals with lower socio-economic status were also less interested in health information [57], they were generally less knowledgeable about their patient rights and less content with their experience of health services [58]. On top of that they have less institutional trust [59]. This underlines that the mandate may affect different population groups differently: The mandate might be more challenging for those with lower socio-economic status, who have less knowledge about the mandate and the vaccine, have more difficulty navigating the health system and find politics less trustworthy. Future evaluation will have to tell whether differences in knowledge persist over time and whether they truly create or exacerbate inequities in that individuals with lower socio-economic status experience more sanctions. Some evidence points to that, for example, within the Australian “no jab, no pay” policy, penalties disproportionately affected parents from vulnerable communities [60], who experienced significant financial loss of the child rebate and limitations to their children’s education opportunities [61, 62] and for whom, additionally, access to vaccination was more difficult [60, 61].

Parents who were more reactant about the measles vaccine mandate rather omitted other vaccines for their children (Hypothesis 2). Also, parents with more reactance about the mandate, indicated lower intention to vaccinate their child against other diseases. This was true even when controlling for vaccine confidence, complacency, constraints, collective responsibility and calculation – established antecedents of vaccination intention. Thus, individuals who are reactant to the mandate might respond to the mandate by reclaiming their freedom by performing a behavior similar to the threatened behavior, i.e. refusing to get vaccinated [23]. This assumed, selective mandates could have potential unintended consequences on vaccination coverage for other vaccines. Thus, our findings confirm prior behavioral experimental research with real-world observational data [24–26]. Contrary to our findings, however, the first available aggregate data from the official reporting system on vaccination coverage for other vaccines (diphtheria, meningococcus C) among infants in 2020 do not confirm this – so far, vaccination coverage has not decreased [51]. Further data will have to validate or refute this finding. A reason for this discrepant finding, if it persists, could be that mandates decrease vaccine uptake for other vaccines among those individuals with high reactance, but have no such effect or even a positive effect on individuals with low reactance. If so, for some parents the visit to the pediatrician to get the mandatory vaccination could also function as a touch-point and be used for further – non-mandatory – vaccinations.

Our data show that the higher the institutional trust, the lower the reactance and, in turn, the more positive the attitude towards the mandate (Hypothesis 3). Institutional trust can be considered as a leverage for a smooth introduction of a mandate. This underlines that vaccine mandates must not be used as the “easy, administrative magic bullet” [63], but that their introduction and implementation must be carefully considered and planned with a view to broader societal (“systemic”) reactions. A mandate is introduced into a complex social and political web characterized by a range of pre-existing (stable or unstable) conditions, concretely with regards to institutional trust, vaccine knowledge and vaccine hesitancy, but also with regards to broader socio-cultural norms and values and attitudes towards science. This points to the need for a multifaceted program to accompany a vaccine mandate and that “maximizes the chances for a ‘friendly reception’ [63]”. Such programs may be able to counter unintended consequences, e.g., by communicating collective responsibility [25] and by building trust in authorities (make process transparent etc.). Attwell et al. compare different strategies to promote consent for a vaccine mandate and find that Australia has successfully appealed to collective responsibility in their communication around the mandate, while France has invested in making processes both transparent and participative as well as providing comprehensive and detailed information, thus building trust in authorities, that should be perceived as competent, objective and sincere [64].

Compared to other studies, a strength of our approach in evaluating the vaccine mandate is that we specifically looked into unintended consequences of a mandate and collected individual behavioral data. Much research on the outcomes of vaccine mandates has focused on vaccination behavior only, using aggregated rather than individual-level data [9, 18]. Against the background of the COVID-19 pandemic, this study may provide valuable insights for policymakers in the ongoing discussions on making COVID vaccination mandatory to increase vaccination uptake. Our findings can help avoid unintended consequences and could be considered already in the preparation phase of a potential upcoming vaccine mandate, e.g., trust in institutions might be a decisive factor determining whether plans to introduce COVID-19 vaccine mandates in different European countries will be successful.

Some limitations to our study need to be acknowledged. Due to quota control we could ensure that the distribution of basic sociodemographic characteristics was largely representative of the distribution of these characteristics among all parents of a youngest child aged 3–18 in Germany. In contrast, no quota control could be achieved for parents of a child aged 0–2 years. The proportion of children in childcare in our data was higher than in official data for children aged 0–2 years (46.0% vs. 34.4%) and, similarly, for children aged 3–5 years (92.2% vs. 91.9%) [65]. Measles protection was comparable, in our data 90.6% of children aged 3–5 years had received at least one measles vaccine dose – compared to 94.4–% of children aged 36 months in official data [51]. The comparable distributions in our data and official data make us confident that our sample did not differ much from the target population. The survey was conducted on behalf of the Robert Koch Institute, the national public health authority in Germany, which might have enhanced social desirability, possibly leading to an overestimation of a favorable attitude towards vaccine mandates and vaccines in general. There might be a selection bias in that those more engaged in the topic, either pro-vaccine or vaccine-hesitant, might have been more likely to participate. This suggests that the observed effects may underestimate the real effects. Participants received a financial incentive to encourage participation and reduce selection bias. We asked parents to answer the survey questions on behalf of their respective youngest child, which might have introduced some bias relevant to measles vaccination status. Parents with several children could be more at ease to vaccinate their youngest child, because it has become routine (or not, because they had negative experiences), they could also experience more constraints, because they have to take care of several children. However, we belief these potential biases have negligible influence on Hypothesis 1–3, as knowledge, reactance and institutional trust were assessed independent of the respective child concerned.

With regards to our results, it has to be emphasized that these are derived from correlational data obtained through a cross-sectional survey at a single point in time and during an interim period, i.e., 6 months after the introduction of the vaccine mandate but prior to the end of the transition period relevant for formal sanctions. We consider a strength of our study, however, is that hypotheses were preregistered and informed by much preliminary work. Further, we developed a DAG (see Supplementary Fig. 1) to define the hypothesized causal pathways for Hypothesis 2. Data collection took place during the COVID-19 pandemic, which might be a confounder. For example, with regards to Hypothesis 1, the pandemic might have influenced the knowledge about the mandate and the measles vaccine (due to lower awareness and lower interest in the new mandate). Moreover, both level of reactance to the mandate and vaccination behavior [66] might have been influenced differently by the pandemic (Hypothesis 2), as well as the level of institutional trust and the attitude towards a vaccine mandate (Hypothesis 3). The pandemic further potentially had an influence on the enforcement of the mandate and on formal sanctions. It remains a challenge to disentangle the effects of the measles mandate from the COVID-19 pandemic, which might have a myriad of effects on vaccine acceptance and vaccine uptake. Nevertheless, we believe that the observed relationships remain valid, although the pandemic influence their strength.

## Conclusion

To conclude, this first study to evaluate the measles vaccine mandate in Germany tells a story of both potentials and challenges. A mandate is best introduced in a society with high institutional trust and trust is something to be worked on continuously. We find that restrictions, such as the mandate, can motivate individuals to reclaim their freedom, i.e., to show a related behavior instead of the sanctioned behavior; such unintended consequences have to be considered whenever restrictive measures are imposed. From an equity perspective, it is evident that a mandate – in order to be fair – has to be accompanied by educative measures to give all citizens equal opportunities to make an informed vaccination decision and has to ensure that vaccination opportunities are easily accessible for all citizens. Policymakers in Germany should now expand communication activities on the mandate, monitor trends in vaccination coverage carefully and take measures to strengthen trust. Furthermore, our study emphasizes that it is important to look beyond vaccination coverage as the sole measure of interest when analyzing immunization programs and to consult social and behavioral science both in the planning and evaluation of vaccine mandates.

## Supplementary Information

**Additional file 1: Supplementary Figure 1. **Directed acyclic graph (DAG) on hypothesis 2: Higher reactance to the measles mandate among parents decreases uptake of other vaccines and the intention to vaccinate children against other diseases. **Supplementary  Table 1.** ANCOVA results for socio-economic status (income) and for knowledge about the measles vaccine mandate and the measles vaccine. **Supplementary Table 2.** ANCOVA results for socio-economic status (education) and for knowledge about the measles vaccine mandate and the measles vaccine. **Supplementary  Table 3.** Parental vaccination decisions for vaccines that had just become due or were soon to come. **Supplementary  Table 4.** Relationship between reactance and vaccination behavior. Results from multiple logistic regression model with hexavalent and pneumococcal vaccination status as outcomes, reactance as predictor and the 5C model, age, gender, region and institutional trust included as covariates. **Supplementary  Table 5.** Relationship between reactance and vaccination intention. Results from multiple linear regression models with intention to get vaccinated against HPV, Tdap, Men C as outcomes, reactance as predictor and the 5C model, age, gender, region and institutional trust included as covariates. **Supplementary  Table 6.** Mediation analyses: Effect of institutional trust (X) on attitude towards the mandate (Y) via reactance (M).

## Data Availability

The data sets used and analyzed during the current study are available from the corresponding author on reasonable request.

## Abbreviations

WHO: World Health Organization
ISCED: International Standard Classification of Education
HPV vaccine: Human papillomavirus vaccine
Tdap vaccine: Tetanus, diphtheria, pertussis vaccine
NITAG: National Immunization Technical Advisory Group
ACME: Average causal mediation effect
M: Mean
SD: Standard deviation
CI: Confidence interval
VIF: Variance inflation factor
AUC: Area under the curve
AIC: Akaike information criterion
DAG: Directed acyclic graph
