# Supplementary material for: How a generally well-accepted measles vaccine mandate may lead to inequities and decreased vaccine uptake: a preregistered survey study in Germany
Source: BMC Public Health. 2022 Oct 3;22:1846. doi: 10.1186/s12889-022-14075-y (PMC9527387; doi:10.1186/s12889-022-14075-y)
Supplement: Supplementary file 1 — Additional file 1: Supplementary Figure 1. Directed acyclic graph (DAG) on hypothesis 2: Higher reactance to the measles mandate among parents decreases uptake of other vaccines and the intention to vaccinate children against other diseases. Supplementary Table 1. ANCOVA results for socio-economic status (income) and for knowledge about the measles vaccine mandate and the measles vaccine. Supplementary Table 2. ANCOVA results for socio-economic status (education) and for knowledge about the measles vaccine mandate and the measles vaccine. Supplementary Table 3. Parental vaccination decisions for vaccines that had just become due or were soon to come. Supplementary Table 4. Relationship between reactance and vaccination behavior. Results from multiple logistic regression model with hexavalent and pneumococcal vaccination status as outcomes, reactance as predictor and the 5C model, age, gender, region and institutional trust included as covariates. Supplementary Table 5. Relationship between reactance and vaccination intention. Results from multiple linear regression models with intention to get vaccinated against HPV, Tdap, Men C as outcomes, reactance as predictor and the 5C model, age, gender, region and institutional trust included as covariates. Supplementary Table 6. Mediation analyses: Effect of institutional trust (X) on attitude towards the mandate (Y) via reactance (M). [file 12889_2022_14075_MOESM1_ESM.docx]

**How a generally well-accepted measles vaccine mandate may lead to inequities and decreased vaccine uptake: a preregistered survey study in Germany**

Julia Neufeind, Nora Schmid-Küpke, Eva Rehfuess, Cornelia Betsch, Ole Wichmann

Table of Contents

[Supplementary Figure 1. Directed acyclic graph (DAG) on hypothesis 2: Higher reactance to the measles mandate among parents decreases uptake of other vaccines and the intention to vaccinate children against other diseases. 3](#_Toc109912033)

[Supplementary Table 1. ANCOVA results for socio-economic status (income) and for knowledge about the measles vaccine mandate and the measles vaccine 4](#_Toc109912034)

[Supplementary Table 2. ANCOVA results for socio-economic status (education) and for knowledge about the measles vaccine mandate and the measles vaccine 5](#_Toc109912035)

[Supplementary Table 3. Parental vaccination decisions for vaccines that had just become due or were soon to come 6](#_Toc109912036)

[Supplementary Table 4. Relationship between reactance and vaccination behavior. 7](#_Toc109912037)

[Supplementary Table 5. Relationship between reactance and vaccination intention. 8](#_Toc109912038)

[Supplementary Table 6. Mediation analyses: Effect of institutional trust (X) on attitude towards the mandate (Y) via reactance (M). 9](#_Toc109912039)

Supplementary Figure 1. Directed acyclic graph (DAG) on hypothesis 2: Higher reactance to the measles mandate among parents decreases uptake of other vaccines and the intention to vaccinate children against other diseases. Lines represent associations between independent and dependent variable and potential confounders.


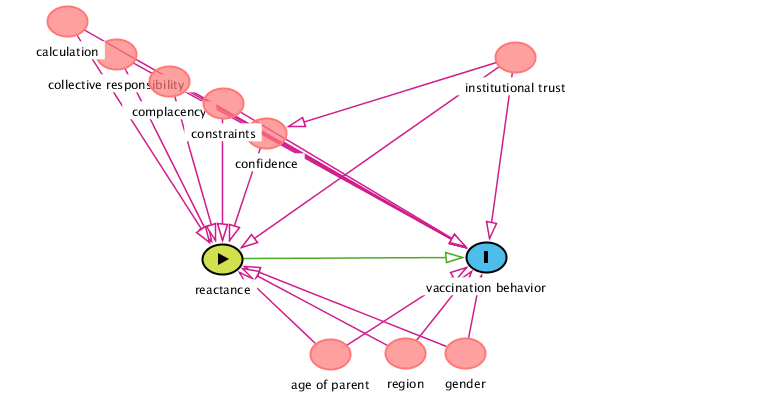


*Note.* The hypothesized associations between calculation, collective responsibility, complacency, constraints, confidence (psychological determinants, 5C-model) and vaccination behavior are described by Betsch et al. (44). Evidence on the influence of institutional trust on vaccination behavior can be found in Neufeind et al. (50). Sprengholz et al. (25) have shown the mitigating role of collective responsibility on vaccination behavior via reactance. The impact of sociodemographic variables such as region or gender have been shown in various studies, (e.g. 51-53, 3).

Supplementary Table 1. ANCOVA results for socio-economic status (income) and for knowledge about the measles vaccine mandate and the measles vaccine

|  | Knowledge about the measles vaccine mandate | | | | | | | |  | Knowledge about the measles vaccine | | | | | | | |
| --- | --- | --- | --- | --- | --- | --- | --- | --- | --- | --- | --- | --- | --- | --- | --- | --- | --- |
|  | df | *F* | *p* | *η_p_^2^* | df | *F* | *p* | *η_p_^2^* |  | df | *F* | *p* | *η_p_^2^* | df | *F* | *p* | *η_p_^2^* |
| Income | 2 | 13.67 | < 0.001 | 0.006 | 2 | 13.99 | < 0.001 | 0.008 |  | 2 | 77.45 | < 0.001 | 0.033 | 2 | 79.16 | < 0.001 | 0.039 |
| Age |  |  |  |  | 1 | 46.16 | < 0.001 | 0.010 |  |  |  |  |  | 1 | 31.82 | < 0.001 | 0.007 |
| Gender |  |  |  |  | 1 | 62.43 | < 0.001 | 0.008 |  |  |  |  |  | 1 | 64.79 | < 0.001 | 0.019 |
| Region (east/west) |  |  |  |  | 1 | 0.27 | 0.601 | 0.060 |  |  |  |  |  | 1 | 6.48 | 0.011 | 0.001 |
| Error | 4520 |  |  |  | 4517 |  |  |  |  | 4520 |  |  |  | 4517 |  |  |  |
| *Note.* Results of ANCOVAs for all preregistered analyses for socio-economic status and knowledge about a) the measles vaccine mandate and b) measles. As predicted, analyses were repeated with age, gender and region (east/west German origin) as control variables. | | | | | | | | | | | | | | | | | |

Supplementary Table 2. ANCOVA results for socio-economic status (education) and for knowledge about the measles vaccine mandate and the measles vaccine

|  | Knowledge about the measles vaccine mandate | | | | | | | |  | Knowledge about the measles vaccine | | | | | | | |
| --- | --- | --- | --- | --- | --- | --- | --- | --- | --- | --- | --- | --- | --- | --- | --- | --- | --- |
|  | df | *F* | *p* | *η_p_^2^* | df | *F* | *p* | *η_p_^2^* |  | df | *F* | *p* | *η_p_^2^* | df | *F* | *p* | *η_p_^2^* |
| Education | 2 | 32.05 | < 0.001 | 0.013 | 2 | 32.82 | < 0.001 | 0.017 |  | 2 | 73.93 | < 0.001 | 0.030 | 2 | 75.28 | < 0.001 | 0.032 |
| Age |  |  |  |  | 1 | 57.36 | < 0.001 | 0.012 |  |  |  |  |  | 1 | 26.00 | < 0.001 | 0.005 |
| Gender |  |  |  |  | 1 | 60.86 | < 0.001 | 0.006 |  |  |  |  |  | 1 | 62.86 | < 0.001 | 0.016 |
| Region (east/west) |  |  |  |  | 1 | 1.44 | 0.23 | 0.000 |  |  |  |  |  | 1 | 2.70 | 0.1 | 0.001 |
| Error | 4860 |  |  |  | 4857 |  |  |  |  | 4860 |  |  |  | 4857 |  |  |  |
| *Note.* Results of ANCOVAs for all preregistered analyses for socio-economic status and knowledge about a) the measles vaccine mandate and b) measles. As predicted, analyses were repeated with age, gender and region (east/west German origin) as control variables. | | | | | | | | | | | | | | | | | |

Supplementary Table 3. Parental vaccination decisions for vaccines that had just become due or were soon to come

|  | Vaccination behavior | | |
| --- | --- | --- | --- |
| Vaccine | Vaccinated  *n* (%) | Not vaccinated *n* (%) | Don’t know  *n* (%) |
| Hexavalent vaccine | 584 (82.8) | 96 (13.6) | 25 (3.5) |
| Pneumococcal vaccine | 494 (70.0) | 121 (17.1) | 90 (12.8) |
|  | Vaccination intention | | |
|  | Rather yes  *n* (%) | Rather no  *n* (%) | Undecided  *n* (%) |
| Men C vaccine | 553 (69.5) | 88 (11.7) | 153 (19.3) |
| HPV vaccine | 1,422 (48.0) | 716 (24.2) | 818 (27.7) |
| Tdap vaccine | 2,475 (83.7) | 195 (6.6) | 286 (9.7) |
| *Note.* Parents with children from two months on to under a year have been asked for their vaccination decisions regarding hexavalent and pneumococcal vaccination. Parents with children under a year have been asked, if they intend to vaccinate against meningococcal disease and parents with children between one and nine years of age have been asked for their vaccination intention regarding human papillomavirus and tetanus, diphtheria, pertussis. | | | |

Supplementary Table 4. Relationship between reactance and vaccination behavior. Results from multiple logistic regression model with hexavalent and pneumococcal vaccination status as outcomes, reactance as predictor and the 5C model, age, gender, region and institutional trust included as covariates. The level of significance is alpha = .05. Significant results are highlighted in bold.

|  | | Hexavalent vaccine | |  | Pneumococcal vaccine | |
| --- | --- | --- | --- | --- | --- | --- |
| Predictors | | *OR* [95% CI] | *OR* [9 % CI] |  | *OR* [95% CI] | *OR* [95% CI] |
| (Intercept) | | 0.77 [0.06, 9.65] | 1.34 [0.09, 18.83] |  | 2.25 [0.20, 25.35] | 1.96 [0.16, 23.99] |
| Reactance | | **0.72 ^**^ [0.57, 0.90]** | **0.70 ^**^ [0.55, 0.89]** |  | **0.67 ^***^ [0.53, 0.85]** | **0.68 ^**^ [0.54, 0.86]** |
| Confidence | | **1.51 ^**^ [1.17, 1.94]** | **1.64 ^***^ [1.23, 2.20]** |  | **1.37 ^**^ [1.08, 1.74]** | **1.35 ^*^ [1.03, 1.77]** |
| Collective responsibility | | 1.14 [0.86, 1.52] | 1.16 [0.86, 1.55] |  | 1.16 [0.87, 1.53] | 1.20 [0.90, 1.60] |
| Constraints | | 1.04 [0.81, 1.35] | 1.06 [0.83, 1.37] |  | 1.08 [0.86, 1.39] | 1.08 [0.85, 1.38] |
| Complacency | | **0.72 ^*^ [0.55, 0.95]** | **0.70 ^*^ [0.53, 0.93]** |  | 0.83 [0.64, 1.09] | 0.82 [0.63, 1.08] |
| Calculation | | 1.10 [0.88, 1.37] | 1.12 [0.89, 1.40] |  | 0.92 [0.75, 1.13] | 0.92 [0.75, 1.13] |
| Age | | 1.03 [0.99, 1.07] | 1.03 [0.99, 1.07] |  | 1.01 [0.97, 1.04] | 1.01 [0.97, 1.05] |
| Gender [w] | | 1.56 [0.89, 2.67] | 1.42 [0.80, 2.45] |  | 1.54 [0.92, 2.57] | 1.49 [0.88, 2.50] |
| Region [west] | | 0.86 [0.44, 1.60] | 0.83 [0.42, 1.55] |  | 0.65 [0.34, 1.16] | 0.63 [0.33, 1.13] |
| Institutional trust | |  | 0.75 [0.53, 1.04] |  |  | 1.01 [0.74, 1.36] |
| Observations | | 671 | 664 |  | 607 | 602 |
| R^2^ Tjur | | 0.174 | 0.189 |  | 0.188 | 0.190 |
| AUC | | 0.847 | 0.848 |  | 0.697 | 0.699 |
| AIC* | | 397.0 | 393.0 |  | 427.6 | 425.3 |
|  | ** p<0.05   ** p<0.01   *** p<0.001* | | | | | |
| *We note that missing cases differ for the models that include institutional trust (variable has additional missing values), therefore comparability of AIC to other models is limited. | | | | | | |

Supplementary Table 5. Relationship between reactance and vaccination intention. Results from multiple linear regression models with intention to get vaccinated against HPV, Tdap, Men C as outcomes, reactance as predictor and the 5C model, age, gender, region and institutional trust included as covariates. The level of significance is alpha = .05. Significant results are highlighted in bold.

|  | HPV vaccine | | | |  | Tdap vaccine | |  | Men C vaccine | | |
| --- | --- | --- | --- | --- | --- | --- | --- | --- | --- | --- | --- |
| Predictors | *B* [95% CI] | | *B* [95% CI] | |  | *B* [95% CI] | *B* [95% CI] |  | *B* [95% CI] | *B* [95% CI] | |
| (Intercept) | 2.63 ^***^ [2.15, 3.10] | | 2.33 ^***^ [1.85, 2.81] | |  | 3.08 ^***^ [2.75, 3.40] | 2.94 ^***^ [2.61, 3.27] |  | 3.82 ^***^ [3.01, 4.63] | 3.81 ^***^ [2.98, 4.64] | |
| Reactance | **-0.16 ^***^ [-0.20, -0.11]** | | **-0.15 ^***^ [-0.20, -0.10]** | |  | **-0.24 ^***^ [-0.28, -0.21]** | **-0.24 ^***^ [-0.27, -0.21]** |  | **-0.28 ^***^ [-0.36, -0.20]** | **-0.28 ^***^ [-0.36, -0.20]** | |
| Confidence | **0.35 ^***^ [0.31, 0.40]** | | **0.29 ^***^ [0.24, 0.34]** | |  | **0.24 ^***^ [0.21, 0.27]** | **0.20 ^***^ [0.17, 0.23]** |  | **0.27 ^***^ [0.19, 0.34]** | **0.27 ^***^ [0.19, 0.36]** | |
| Collective Responsibility | -0.00 [-0.06, 0.05] | | 0.00 [-0.05, 0.06] | |  | **0.10 ^***^ [0.06, 0.14]** | **0.10 ^***^ [0.07, 0.14]** |  | 0.02 [-0.08, 0.12] | 0.03 [-0.07, 0.13] | |
| Constraints | **0.05 ^*^ [0.01, 0.10]** | | **0.05 ^*^ [0.01, 0.09]** | |  | 0.03 [-0.00, 0.06] | 0.02 [-0.00, 0.05] |  | 0.05 [-0.02, 0.12] | 0.05 [-0.02, 0.12] | |
| Complacency | **-0.08 ^**^ [-0.13, -0.02]** | | **-0.07 ^**^ [-0.13, -0.02]** | |  | **-0.14 ^***^ [-0.18, -0.11]** | **-0.14 ^***^ [-0.18, -0.10]** |  | -0.09 [-0.18, 0.00] | **-0.10 ^*^ [-0.19, -0.00]** | |
| Calculation | -0.02 [-0.06, 0.01] | | -0.03 [-0.07, 0.00] | |  | **0.08 ^***^ [0.05, 0.10]** | **0.07 ^***^ [0.04, 0.09]** |  | 0.02 [-0.03, 0.08] | 0.02 [-0.04, 0.08] | |
| Gender [w] | **0.11 ^*^ [0.02, 0.20]** | | **0.13 ^**^ [0.04, 0.22]** | |  | **0.19 ^***^ [0.12, 0.25]** | **0.20 ^***^ [0.13, 0.26]** |  | **-0.17 ^*^ [-0.33, -0.01]** | **-0.19 ^*^ [-0.35, -0.02]** | |
| Age | -0.00 [-0.01, 0.00] | | -0.00 [-0.01, 0.00] | |  | **0.01 ^***^ [0.00, 0.01]** | **0.01 ^***^ [0.00, 0.01]** |  | -0.01 [-0.02, 0.01] | -0.01 [-0.02, 0.01] | |
| Region [west] | -0.06 [-0.16, 0.04] | | -0.08 [-0.17, 0.02] | |  | 0.02 [-0.05, 0.09] | 0.01 [-0.06, 0.08] |  | -0.11 [-0.28, 0.05] | -0.12 [-0.29, 0.05] | |
| Institutional trust |  | | **0.16 ^***^ [0.11, 0.22]** | |  |  | **0.09 ^***^ [0.05, 0.13]** |  |  | -0.02 [-0.12, 0.07] | |
| Observations | 2872 | | 2860 | |  | 2872 | 2860 |  | 781 | 774 | |
| *R*^2^ / *R*^2^ adjusted | 0.202 / 0.200 | | 0.211 / 0.209 | |  | 0.425 / 0.423 | 0.428 / 0.426 |  | 0.278 / 0.270 | 0.284 / 0.275 | |
| AIC | 8674.2 | | 8609.4 | |  | 6515.8 | 6461.7 |  | 2134.8 | 2114.1 | |
|  | |  | | ** p<0.05   ** p<0.01   *** p<0.001* | | | | | | |  |
| **We note that missing cases differ for the models that include institutional trust (variable has additional missing values), therefore comparability of AIC to other models is limited.* | | | | | | | | | | |  |

Supplementary Table 6. Mediation analyses: Effect of institutional trust (X) on attitude towards the mandate (Y) via reactance (M). The level of significance is alpha = .05. Significant results are highlighted in bold.

|  | Mediator variable model (outcome: reactance) | | |  |
| --- | --- | --- | --- | --- |
| Predictor | *B* | SE | 95% CI | *p* |
| Constant | 2.81 | 0.06 | [2.69, 2.93] |  |
| Institutional trust | **-0.30** | **0.02** | **[-0.34, -0.26]** | **<.001** |
| Institutional trust (adjusted) | **-0.30** | **0.02** | **[-0.34, -0.27]** | **<.001** |
|  | Dependent variable model (outcome: attitude towards mandates) | | | |
|  | Model summary: *R*^2^ = 0.29 | | |  |
| Predictor | *B* | SE | 95% CI | *p* |
| Constant | 4.33 | 0.04 | [4.24, 4.41] |  |
| Institutional trust | **0.21** | **0.01** | **[0.19, 0.23]** | **<.001** |
| Reactance | **-0.61** | **0.01** | **[-0.63, -0.59]** | **<.001** |
| Institutional trust (adjusted) | **0.21** | **0.01** | **[0.19, 0.23]** | **<.001** |
| Reactance (adjusted) | **-0.61** | **0.01** | **[-0.63, -0.59]** | **<.001** |
|  | Indirect effect of X on Y via reactance | | |  |
| Mediator | *B* |  | 95% Quasi-Bayesian CI | *p* |
| Reactance | **0.18** |  | **[0.16, 0.21]** | **<.001** |
| Reactance (adjusted) | **0.19** |  | **[0.17, 0.21]** | **<.001** |
| *n* = 4,704 | | | | |
|  |  |  |  |  |
